# Bainbridge-ropers syndrome caused by loss-of-function variants in ASXL3: Clinical abnormalities, medical imaging features, and gene variation in infancy of case report

**DOI:** 10.1186/s12887-020-02027-7

**Published:** 2020-06-09

**Authors:** Linfeng Yang, Bin Guo, Weiwei Zhu, Lei Wang, Bingjuan Han, Yena Che, Lingfei Guo

**Affiliations:** 1 Jinan Maternal and Child Care Hospital, No.2, Jianguo xiao jing-san Road, Jinan, 250001 Shandong Province China; 2 Jinan Central Hospital Affiliated to Shandong University, No. 105, Jiefang Road 250013, Jinan, 250011 Shandong Province China; 3 Department of MRI, Shandong Medical Imaging Research Institute, Cheeloo College of Medicine, Shandong University, Jing-wu Road No. 324, Jinan, 250021 Shandong Province People’s Republic of China

**Keywords:** ASXL3 gene, Bainbridge–ropers syndrome, Psychomotor retardation, Magnetic resonance imaging, Whole-exome sequencing

## Abstract

****Background**:**

Bainbridge–Ropers syndrome (BRPS) is a recently described developmental disorder caused by de novo truncating mutations in the Additional sex combs-like 3 (ASXL3) gene. Only four cases have been reported in China and are limited to the analysis of its clinical abnormalities, medical imaging features and gene variation. The aim of this study was to investigate the clinical phenotype, imaging manifestations and genetic characteristics of BPRS syndrome caused by ASXL3 gene mutation. Clinical data, medical imaging data and gene test results of BRPS in infant patients were retrospectively analyzed, and related literature was summarized.

****Case presentation**:**

At the age of 8 months, brain MRI showed that the subarachnoid space of the forehead was widened, part of the sulci was deepened, and the corpus callosum was thin. The development quotient (DQ) was determined using the 0~6-year-old pediatric examination table of neuropsychological development at 6 months and 8 months. The DQ of both tests was less than 69. Whole-exome sequencing revealed a heterozygous frameshift mutation c.3493_3494deTG in exon 12 of the ASXL3 gene, resulting in the amino acid change p. (Cys1165Ter). No variation was present at this site in her parents. Sanger sequencing of family members validated this analysis, suggesting a de novo mutation. The de novo ASXL3 mutations generated stop codons and were predicted, in silico, to generate a truncated ASXL3.

****Conclusions**:**

The main clinical features of the patient included psychomotor development retardation, difficulty in feeding, hypotonia, and special facial features. MRI features showed that brain development lagged behind that of normal children. Genetic testing is helpful in the early diagnosis of BRPS.

## Background

Bainbridge–Ropers syndrome (BRPS; OMIM:615485) was first described in 2013 and is characterized by failure to thrive, feeding problems, hypotonia, intellectual disability (ID), autism, postnatal growth retardation, abnormal facial features with arched eyebrows, anteverted nares and delays in language acquisition [1]. A reverse-genetics approach has suggested proven particularly important for the discovery of disorders such as BRPS, whose main clinical features are nonspecific, especially when looked at in isolation or in a small number of patients. De novo mutation status is the first clue to the potential pathogenicity of a given variant, and such mutations are known to constitute a significant proportion of the underlying causes of moderate and severe ID [2].

BRPS is caused by de novo dominant truncating variants in the transcriptional regulator gene Additional Sex Combs Like 3 (ASXL3), and missense variants in ASXL3 have been identified in individuals with autism spectrum disorder (ASD) [3–5]. ASXL family members are assumed to be epigenetic regulators that are involved in hereditary neurological disorders as well as malignancies [6–8]. Truncating mutations in ASXL1 have been reported in association with Bohring-Opitz syndrome (BOS), which has phenotypic overlap with BRPS [9]. Both, ASXL1 and ASXL3, are expressed in tissues like brain, spinal cord, kidney, liver and bone marrow, but ASXL3 is expressed a lower level [10]. The high correlation of expression patterns between ASXL1 and ASXL3 may account for some of the shared phenotypic features.

BRPS is very rare worldwide; over 30 cases of BRPS have been reported abroad, and four cases have been reported in China, which were limited to the analysis of the clinical characteristics and genetic characteristics [6, 11]. We report a case of BRPS in Shandong Province and describe the clinical abnormalities, medical imaging characteristics and genetic characteristics of this disease through a literature review to improve the comprehensive understanding of this disease for pediatricians and radiologists.

## Case presentation

The patient (female, 6 months) was treated for “psychomotor retardation for more than 3 months”.

The patient mother was primiparous and had been pregnant at the age of 29 years. She denied any exposure to poisons, chemicals and radiation during pregnancy and regular prenatal examinations. At 36 weeks, the patient was delivered by cesarean section due to “breech and intrauterine distress” (poor fetal heart monitoring response), and her crying was weak 1.5 h after birth. There was no premature rupture of membranes, no history of postnatal asphyxia, and no abnormality in amniotic fluid, placenta or umbilical cord. Her Apgar score was 10 points 1 min and 5 min after birth, and she weighed 2.9 kg at birth.

**Fig. 1:**
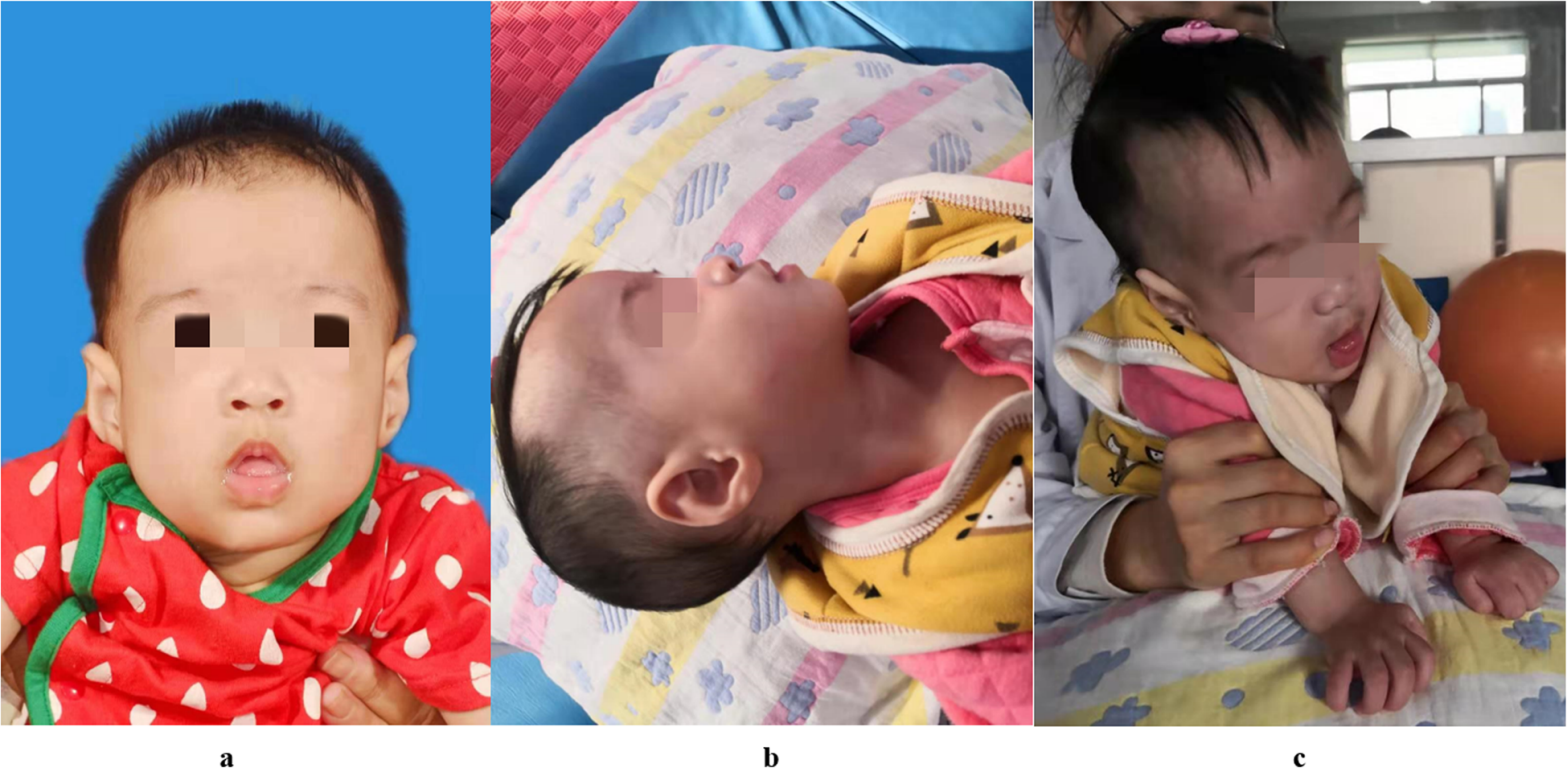
**a-c** Prominent forehead, protruding superior orbital crest, strabismus, nose unprecedentedly tilted, middle flat, lower lip eversion, and small jaw **a, b**. Both hands were clawed, no clenched fists, wrists sagged, and both wrists slightly ulnar when laid flat **c**

At the age of 6 months, the child could not lift her head, and the rehabilitation treatment was performed for 1 month with poor effect. Neuropsychological tests were performed at the age of 6 months, indicating overall backward development, and she was admitted to the hospital for examination. Admission examination: 70 cm height (normal value at the same age, 67.3 cm), 6.4 kg weight (normal value at the same age, 7.6 kg), 45 cm head circumference (normal value at the same age, 42.8 cm), backward development, malnutrition, general mental reaction, normal skin color, no yellow dye, large and round bilateral pupil, no sensitivity to light reflection on left side, can pursue things, and response to sound. Prominent forehead, protruding superior orbital crest, strabismus, nose unprecedentedly tilted, middle flat, lower lip eversion, and small jaw were observed (Fig. 1). Breath was a little short, double lung breath sound was thick, and cardiac auscultation was normal. Abdomen was smooth and soft; subcutaneous fat was thin; liver, spleen and ribs were not touched; the whole body muscle strength, muscle tension was low; could not raise head and lift the chest while lying on the stomach; fingers were slender; flexion in both hands was clonic; could not make a fist; pendulous wrist; and the wrist had ulnar flexion. Physiological reflex existed, but pathological reflex was not induced.

The parents of the patient were in good health and deny inbreeding and reproduction of similar disabled patients in their family.

### Medical imaging

The patient underwent brain MRI to assess brain development. In addition, X-ray examination of both the lower limbs and chest was performed to evaluate bone development. All MRI was performed using a 1.5-T MR scanner (Achieva, Philips Medical Systems) and a 16-channel phased array head coil with T2WI, T1WI, T2-FLAIR, DWI sequences with a field of view (FOV) of 230 × 190 mm^2^, slice thickness of 5 mm, gap of 0.5 mm, axial scanning, and T2WI sagittal sections. The scan time was approximately 10 min. Each image was evaluated by a senior neurologist who was unaware of the established diagnosis. There are several reported scoring schemes for the severity of cortical atrophy and ventricular dilation, such as the semiquantitative ten-point scale devised by Yue et al. [12] and the ARWMC rating scale by Wahlund LO [13]. After reference to the evaluation criteria proposed in the above two documents, the neuroradiologist used a simpler scheme and scored the findings as mild, moderate or severe based on gross visual assessment.

### Genetic tests and laboratory examination

After informed consent was obtained from the parents, 2 ml of peripheral blood was extracted from the child and her parents, and total exon target region capture and high-throughput sequencing were used for detection. The patient was screened for hematuria and genetic metabolic disease.

### Neuropsychological scores

The neuropsychological test was the 0~6-year-old pediatric examination table of neuropsychological development (Institute of Pediatrics of Capital Medical University, CNBSR 2016). Children are considered to have a developmental concern if their development quotient (DQ) in any specific domain is ≤69. The following classification standard was used: ≥130, excellent; 115–129, intelligent; 84–114, normal; 70–84, lower than normal and ≤ 69, low.

**Fig. 2:**
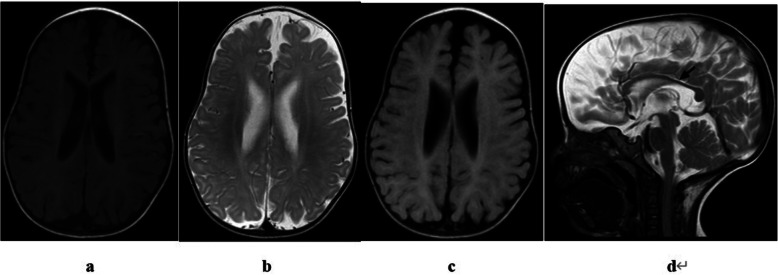
**a-d** The imaging findings were mainly mild lateral ventricular enlargement **a-c**. The corpus callosum thinned (**d**, black arrow), and the subarachnoid space widened

**Fig. 3:**
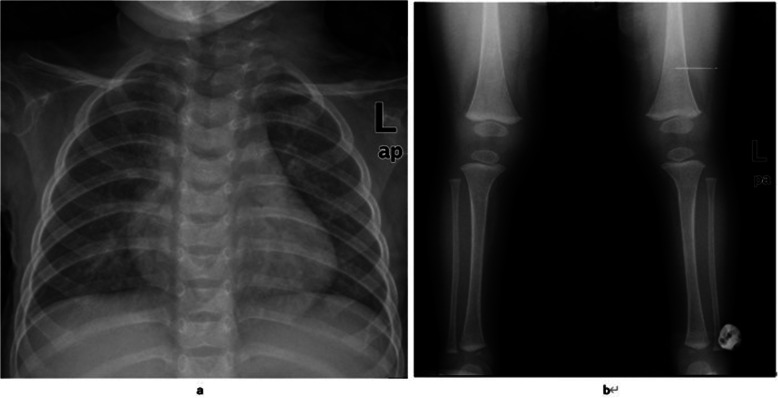
**a, b** Both chest and lower limbs showed no obvious abnormalities

At the age of 8 months, brain MRI showed that the subarachnoid space of the forehead was widened, part of the sulci was deepened, the corpus callosum was thin, no significant high signal was observed in the white matter of the brain, and no significant abnormal high signal was observed on DWI (b = 1000). A detailed assessment of the MRI results is shown in Table 1 and Fig. 2. X-ray examination of both the lower limbs and chest is shown in Fig. 3. Electroencephalogram (EEG) was normal, with no epileptic discharge. A bilateral adrenal CT scan showed no obvious abnormality.

**Table 1:** The MRI findings, neuropsychological development score, genetic analysis report of BRPS infancy patients

The cerebral MRI findings													
Age	cerebral sulcus widened	Cerebellar atrophy	Brainstem thinning	Corpus callosum thinning	Cortical atrophy (sulcal widening)	Ventricular dilation(0–3)	Subcortical white matter change	Periventricular white matter change	Myelination abnormality	Internal capsule change	Signal change consistent with focal infarct	Abnormal signal in basal ganglia	Other findings
8 months after birth	+	–	–	+	+	1	–	–	–	–	–	–	–
**The examination table of neuropsychological development**													
**Date**	**Big activity sport**	**Fine sport**		**Adaptive capacity**		**Language capacity**	**Social behavior**		**Development quotient**				
6 months after birth	48.4	40.3		56.5		48.4	32.3		45.2				
8 months after birth	51.3	38.5		57.7		25.6	25.6		39.7				
**Genetic analysis report**													
**Gene**	**Chromosomal location**	**Transcript number**	**exon**	**Nucleotide amino acid**		**• Homozygous/heterozygous**	**Normal frequency**	**Pathogenicity analysis**	**inheritance mode**		**Disease/phenotype**		**Source of variation**
**ASXL3**	chr18:31323304–31,323,306	NM_030632	exon12	c.3493—3494 del (p.C1165fs)		het	_	pathogenic	AD		Bainbridge–Ropers syndrome		Spontaneity

The DQ was determined using the 0~6-year-old pediatric examination table of neuropsychological development (CNBSR 2016) at 6 months and 8 months, as shown in Table 1. The DQ of both tests was less than 69.

Routine test results for hematuria and feces, liver and kidney function, electrolytes and myocardial enzymes were normal. There were no abnormalities in thyroid function and no abnormalities in blood ammonia and lactic acid. There were no abnormalities in parathyroid hormone, 25-hydroxyvitamin D or blood tandem mass spectrometry.

**Fig. 4:**
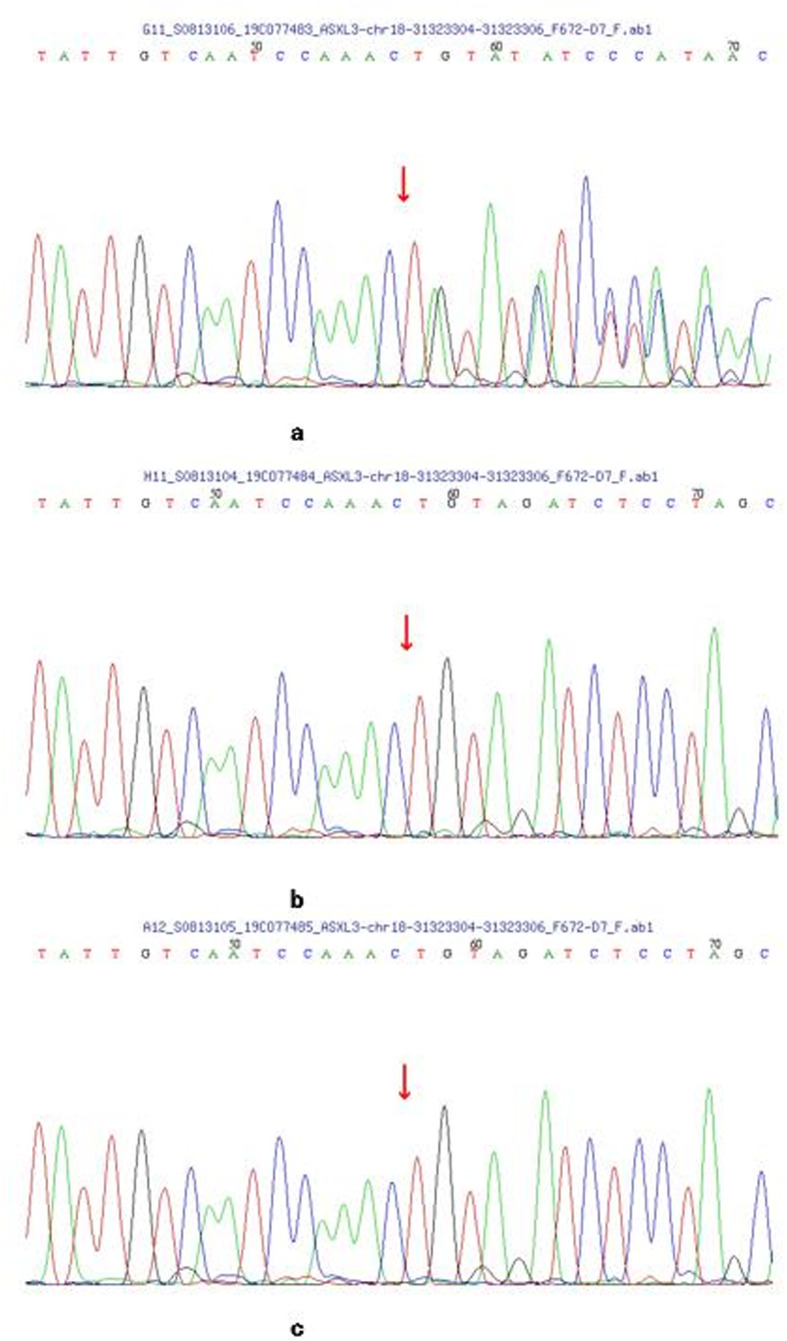
**a-c** Sanger sequencing of the ASXL3 mutation in the patient and her parents. **a**, A heterozygous frame shift mutation (**c**.3493_3494del) in the patient. **b**, Not found in her father. **c**, Not found in her mother. The arrow indicates the location of the mutation

Whole-exome sequencing revealed a heterozygous frameshift mutation c.3493_3494deTG in exon 12 of the ASXL3 gene, resulting in the amino acid change p.(Cys1165Ter). No variation was present at this site in her parents. Sanger sequencing of family members validated this analysis, suggesting a de novo mutation (Fig. 4). The de novo ASXL3 mutations generated stop codons and were predicted, in silico, to generate a truncated ASXL3. These variants are found at low frequency within healthy control populations. The predicted results of SIFT, PolyPhen-2, Mutation Taster, GERP++, and REVEL were unknown. According to the 2015 ACMG Guidelines [14], the c.3493_3494del mutation is pathogenic.

## Discussion and conclusions

ASXL3, the pathogenic gene of rare BRPS, is a member of the ASXL gene family, located at 18q12.1, with a length of 6.8 kb, containing 12 exons and encoding the multicomb protein. The polycarpus family is a group of transcription factors that regulate target genes at the chromatin level through epigenetic modification, and they are involved in embryonic development, cell proliferation, and tumor formation [15]. Missense variations in the ASXL3 gene are closely associated with autism spectrum disorders, and dysfunction variants can lead to severe psychomotor retardation [16, 17]. BRPS and Bohring-Opitz syndrome (BOS) are both genetic diseases with ASXL gene abnormalities [18], which are difficult to distinguish in clinical practice and can manifest as severe psychomotor retardation, difficulty in feeding, hypotonia and microcephaly [19, 20]. With the increase in cases of BRPS syndrome reported in recent years, the clinical manifestations of the two diseases have also been found to be different. BOS syndrome is usually accompanied by intrauterine growth restriction, with typical special phenotypes, such as triangular head, eyeball protrusion, facial bright red spot mole, upper eyelid oblique and elbow flexion [20, 21]. Although the child in this case was born by cesarean section at 36 weeks due to “breach-position and intrauterine distress”, she did not have the above special face after birth (Fig. 1). In our case, WES revealed a de novo heterozygous frameshift variant in exon 12 of the ASXL3 gene [chr18:31323305-31323306delTG: c.3493_3494del TG, p.(Cys1165Ter)]. The amino acid change in codon 1165 of ASXL3 was previously reported in a Russian female by Kuechler et al. [chr18:g.31,323,306_31,323,307delGT, c.3494_3495delGT, p.(Cys1165Ter)] [22]. Although the two mutations have different chromosomal locations, they both cause the protein to terminate at the same site. In the previous literature, it was reported that a Russian patient was close to the gene mutation site in this study and had facial symptoms similar to those in this study. The Russian girl showed abnormally clenched hands with ulnar deviation and a high palate. Her face was long with a prominent forehead, temporal narrowing, down slanting palpebral fissures, prominent columella and small alae nasi and downturned corners of the mouth. She had long and slender hands/fingers, still with some intermittent ulnar deviation, muscular hypotonia of the trunk and rather hypertonic extremities. The comparison of the clinical manifestations of the two patients indicates that there may be some correlation between similar gene mutation sites and clinical manifestations.

The clinical phenotype of BPRS is complex. Among the more than 30 patients with detailed clinical data worldwide, in addition to psychomotor retardation and severe language impairment, they also presented with multiple symptoms. Even though they carry the same ASXL3 gene mutation sites in patients, the clinical characteristics also differ; Srivastava [16] and Balasubramanian [6] reported that they found the ASXL3 gene c.4330C > T nonsense mutations in two patients; one patients has fetal intrauterine growth restriction, feeding difficulties, convulsions, and loss of language, while the other patient does not have these symptoms and can speak simple words. The child in our research was born by cesarean section at 36 weeks due to “breach-position and intrauterine distress”. When she came to the hospital, her height were normal, but her weight was lower than normal. After birth, the child had poor sleep, easy waking, high muscle tension, and difficulty feeding, suggesting that the child may have suffered from vegetative nerve dysfunction before and after birth.

During the treatment of this patient since admission, we always used the DQ test to evaluate the developmental status of the patient. The neuropsychological test was the 0~6-year-old pediatric examination table of neuropsychological development. According to the current two tests, the DQ value of the patient was significantly lower than the normal value, as shown in Table 1. There was no significant change or increase in the two measurements of the DQ value. We will follow up the test in future treatment and see the correlation between changes in brain MRI performance and changes in DQ.

In the previous literature, relatively complete brain MRI data of three patients were presented, with the primary understanding of brain characteristics of BRPS patients based on MRI data. Mild white matter reduction, thinner corpus callosum, mild lateral ventricle enlargement and cerebellar vermis dysplasia were the main manifestations of MRI results in the three patients. One of the patients also underwent a scan of the susceptibility weighted imaging sequence, showing an irregular hypointensity in the right frontal lobe. The imaging findings of the patient in this study were mainly mild lateral ventricular enlargement. The corpus callosum thinned, and the subarachnoid space widened, which are similar to the imaging findings in the previous literature, but there were no obvious abnormal signals in the brain parenchyma, as shown in Fig. 2. In future follow-up, MRI functional sequence scanning should be added to understand changes in brain structure, oxygen metabolism, water molecule movement and other aspects of patients.

There are few reports of BRPS in China, and it is necessary to collect more cases and increase the ASXL3 gene mutation spectrum of children with BRPS in China to search for the relationship between genotypic and clinical characteristics. Most children show moderate to severe mental disabilities and severe language impairment, and some children have autism, epilepsy, etc. [6]. The prognosis of BPRS patients is poor. There is no effective treatment for BPRS, so only symptomatic treatment can be conducted. For example, rehabilitation treatment for psychomotor retardation in the early stage is conducted, and the specific effect needs to be verified by long-term follow-up data. Clinical features of BPRS like feeding difficulties, growth disorders, developmental delays and special faces of children, should be identified as soon as possible to improve the genetic examination and clear diagnosis.

The main clinical features of the patient included psychomotor development retardation, difficulty in feeding, hypotonia, and special facial features. MRI features showed that brain development lagged behind that of normal children. The mutation site in the ASXL3 gene in this patient was first reported in China, and the mutation in the ASXL3 gene c.3393_3494 > T was the pathogenic cause of BRPS this child. The pathogenic variation in the ASXL3 gene was new and functionally deficient.

## Abbreviations

BRPS: Bainbridge–Ropers syndrome
ASXL3: Additional sex combs-like 3
DQ: Development quotient
ID: Intellectual disability
FOV: Field of view
EEG: Electroencephalogram
BOS: Bohring-Opitz syndrome
